# Acute Management of Iatrogenic Injury to Vertebral Artery With Central Venous Catheter in a Critically Ill Patient

**DOI:** 10.7759/cureus.9956

**Published:** 2020-08-23

**Authors:** Aisha Al Rayes, Yasir Khattak, Avdullah Qafani, Muhammad Anwar, Ayman Al Sibaie

**Affiliations:** 1 Vascular Surgery, Dubai Medical College, Dubai, ARE; 2 Radiology, Rashid Hospital, Dubai, ARE; 3 Vascular Surgery, Rashid Hospital, Dubai, ARE

**Keywords:** endovascular repair, vertebral artery injury, iatrogenic injury, misplaced central venous catheter

## Abstract

Vertebral artery (VA) injury during catheterization is quite rare given its anatomical position, but can be catastrophic when it is not discovered early on and managed accordingly. A multidisciplinary approach to the management of such injury has to weigh-in the benefits and risks of open surgery versus endovascular intervention. This can be done after thorough assessment of the patient’s condition and accessibility of the injured vessel. We report a case of a 90-year-old female admitted as a case of pneumonia associated with decreased level of consciousness. She acquired an iatrogenic injury due to insertion of central venous catheter (CVC) into her dominant right VA as confirmed via CT angiography (CTA). This case report aims to highlight the role of endovascular intervention in the acute management of VA injury in a critically ill patient.

## Introduction

Injury to the vertebral artery (VA) is not a common occurrence, but can occur during invasive procedures performed in the head and neck region; one of which being central venous catheter (CVC) insertion, with an incidence of <1% [1]. An early clinical suspicion and diagnosis can prompt immediate management and in turn avoid complications such as stroke, lacerations, dissection, hemorrhage, fistula formation, and rapidly growing pseudo-aneurysm leading to obstructive symptoms [2-3]. This type of injury is not commonly reported in the literature, hence the lack of treatment guidelines. Nonetheless, the minimally invasive nature of endovascular procedures, and favorable clinical outcome, prove it to be a safer first line treatment of choice for uncomplicated VA injury rather than open surgery.

## Case presentation

A 90-year-old female was admitted to our hospital as a case of pneumonia associated with decreased level of consciousness; Glasgow Coma Scale (GCS) was 9/15 which deteriorated days later to 5/15. Along the course of her admission, the patient was admitted in the ICU and later shifted to high dependency unit (HDU) where she remained under close observation. There, she became vitally unstable, and had an episode of apnea and asystole for which cardiopulmonary resuscitation (CPR) was started immediately as per ACLS guidelines. Right internal jugular catheter was inserted using the conventional landmark technique but subsequent aspiration revealed bright color of blood and arterial blood gas (ABG) analysis was suggestive of arterial blood. A STAT dose of clexane 40 mg was administered to prevent risk of thrombosis and catheter was left in situ until confirmation of the site of entry.

**Figure 1:**
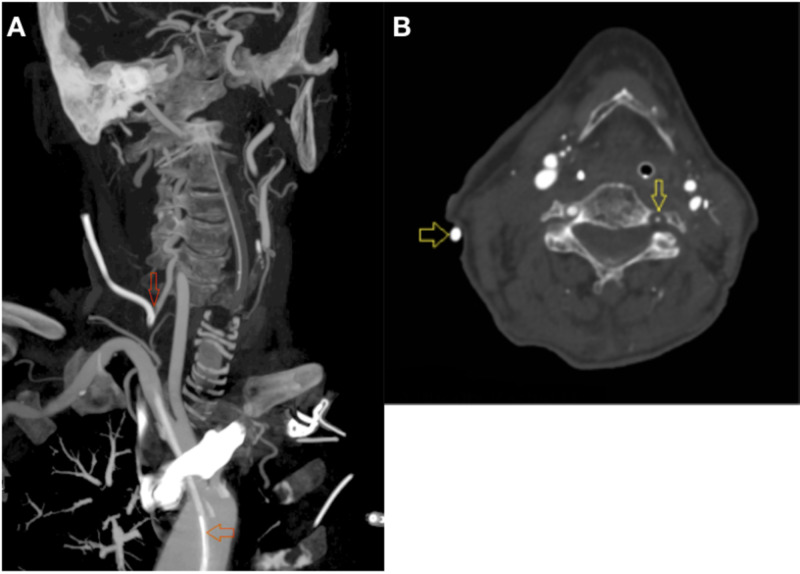
(A) Entry site of CVC into the VA (narrow arrow). CVC within the descending thoracic aorta (wide arrow). (B) Hypoplastic VA (narrow arrow). CVC (wide arrow). CVC, central venous catheter; VA, vertebral artery

**Figure 2:**
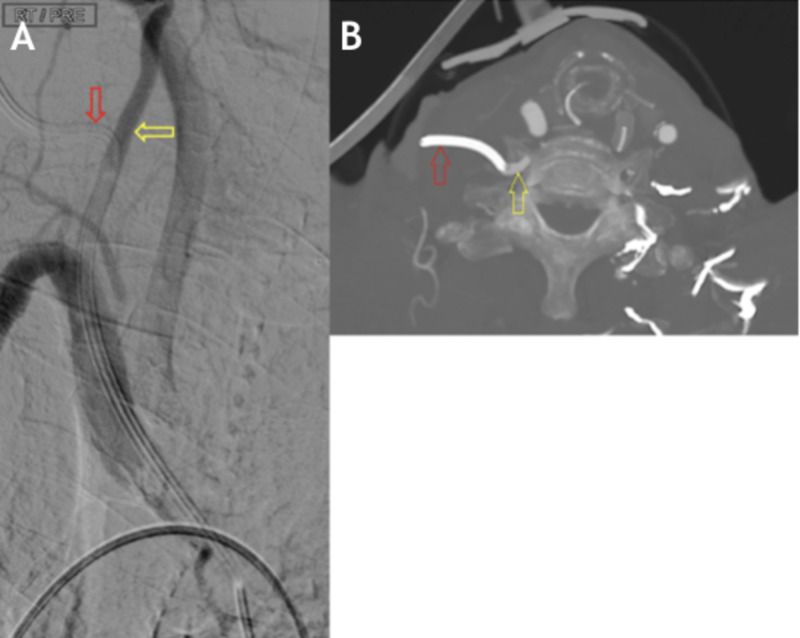
(A & B) Point of central line entry (red arrow) into the VA (yellow arrow). VA, vertebral artery

Bedside carotid ultrasound assessment was not conclusive as the catheter was not seen in the carotid artery. CT angiography (CTA) confirmed the entry of the catheter in the right VA, extending caudally into the subclavian and further into the brachiocephalic trunk, arch of aorta and further up to the descending aorta. In addition, the left VA appeared hypoplastic with nonfilling of the artery beyond the V4 segment representing posterior inferior cerebellar artery continuation (Figures 1-2). 

The risks and benefits of both surgical and endovascular intervention were discussed. In view of the patient’s general condition and recent episode of resuscitation, surgical intervention carried the highest risk of complications. With the involvement of the dominant VA the possibility of stroke was also higher with surgical approach. Considering these factors endovascular management was chosen as the preferable treatment option in this case, both by the vascular and interventional radiology team.

**Figure 3:**
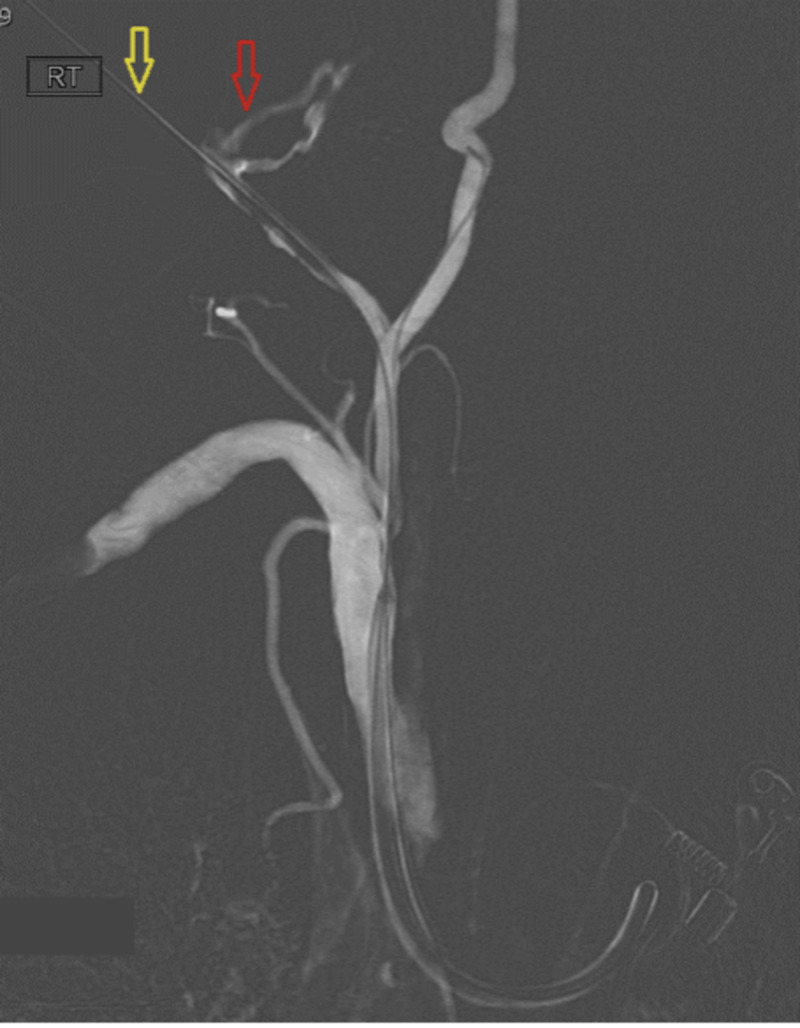
Active extravasation of contrast (red arrow) following partial withdrawal of the CVC over a safety wire (yellow arrow). CVC, central venous catheter

Following general anesthesia and sterile preparation of the right groin, access was achieved into the right femoral artery and 5 Fr sheath was placed. Some 5000 IU of heparin was injected through the femoral sheath to prevent thrombotic complications. Some 5 Fr vertebral catheter was used for cannulation of right VA. Angiographic images were acquired in multiple projections. Iatrogenic injury of the right VA was known from the previous CT scan. A 0.035’’ safety Glidewire (Terumo Medical Corporation, Tokyo, Japan) was placed through the central line. The catheter was partially pulled over the wire and angiogram performed, which confirmed and localized the site of active extravasation from the entry site of CVC into the V1 (preforaminal) segment of VA (Figure 3). Good flow of contrast was noted beyond the site of extravasation with complete opacification of the basilar artery and posterior circulation.

The 5 Fr sheath was then replaced with a Neuron Max 0.88 Soft tip 6 Fr long sheath (Penumbra, Inc., Alameda, CA, USA) and access achieved into the right brachiocephalic trunk. A Navien 0.72 intermediate catheter was placed via the Neuron Max and positioned at the ostium of the VA.

**Figure 4:**
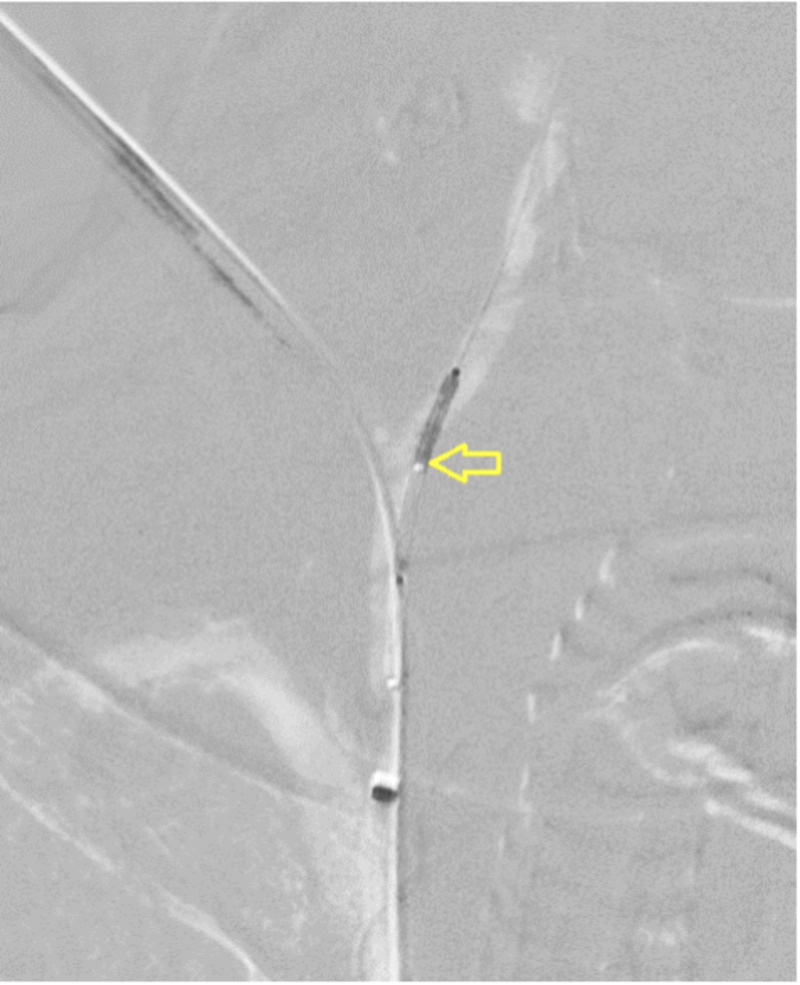
Stent graft (arrow) positioned at the entry site of CVC. CVC, central venous catheter

**Figure 5:**
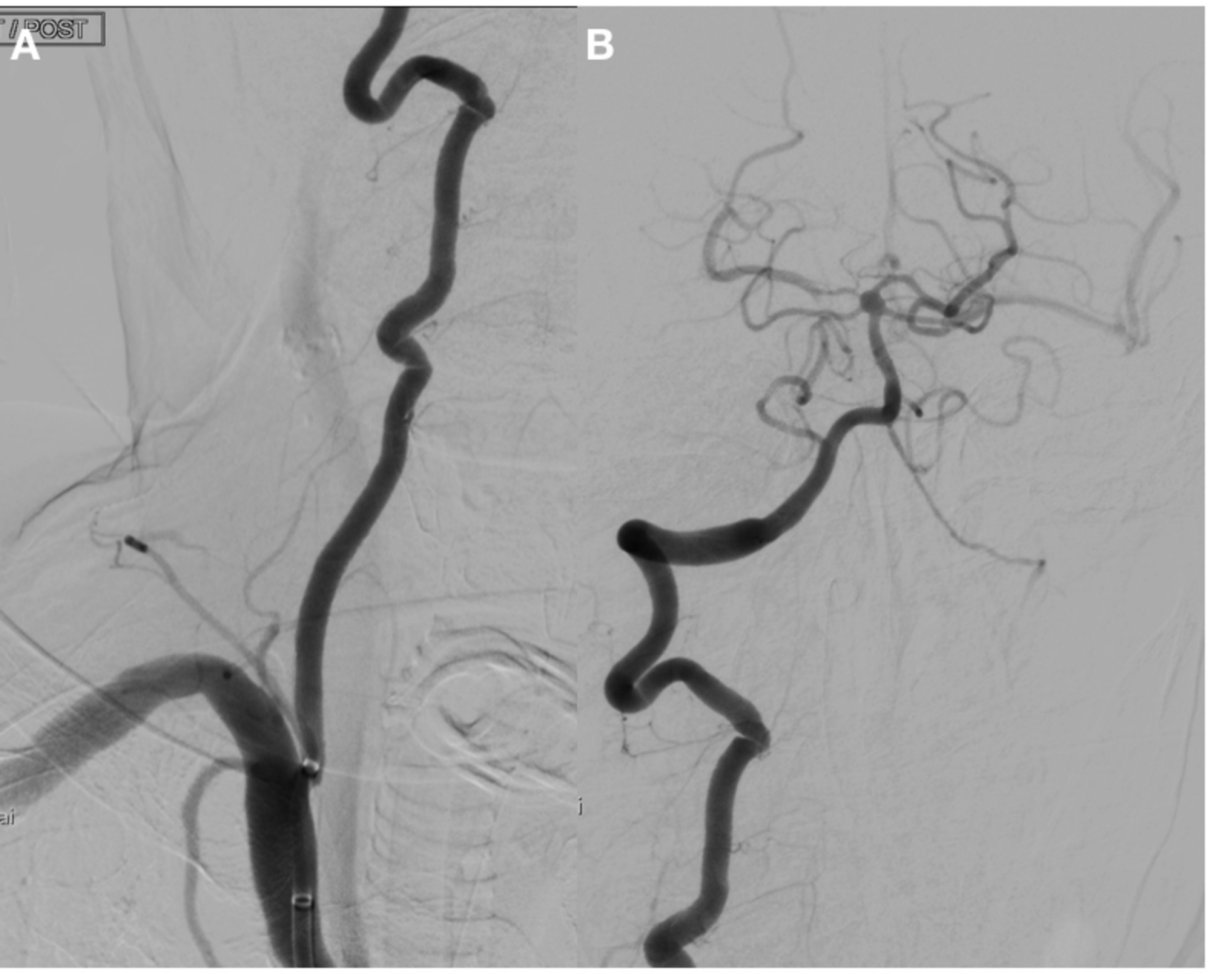
(A) Post stent graft deployment. Good vessel wall reconstruction noticed with no extravasation of contrast at the iatrogenic injury site. (B) Good flow into the intracranial VA with filling of the basilar artery and its branches. VA, vertebral artery

A 0.014 balance middle weight wire (Abbott Vascular, CA, USA) access was then achieved into the VA beyond the site of extravasation. A balloon expandable Bentley stent graft (Bentley InnoMed GmbH, Hechingen, Germany) measuring 5 mm in diameter and 1.8 cm in length was positioned at the site of arterial injury (Figure 4). Central line was removed at this point of time and the graft deployed. Angiogram performed following graft placement showed good vessel wall reconstruction with complete exclusion of extravasation (Figure 5). As per the hospital's departmental protocol, femoral sheaths are removed by the end of the procedure before the patient is shifted out of the catheterization lab, provided the diameter is not bigger than 9 Fr. If greater than 9 Fr a ProGlide closure device is used for hemostasis. In this case, vascular sheath was removed and hemostasis secured using manual compression. No immediate postprocedural complications were noted.

## Discussion

A penetrating injury such as an iatrogenic VA cannulation can be asymptomatic or stable at presentation in around 74% of patients, but can carry fatal consequences if remained undiagnosed, hence the importance of early diagnosis and treatment [4]. The deep position of the VA poses a management dilemma to the surgeon when faced by an injury in that anatomical location. Different approaches to the management of inadvertent arterial cannulation were mentioned in the literature including pull/pressure technique, surgical, and endovascular.

One of the methods described is the pull/pressure technique, which may only be effective if the artery is accessible to manual compression. Guilbert et al. [5] associated this technique with a higher risk of developing hematoma, airway obstruction, immediate stroke (5.6%), false aneurysm, and a significantly higher morbidity as compared to surgical and endovascular management. Shah et al. [3] reported two patients treated with this technique; one patient developed a stroke and died, the second patient developed a pseudo-aneurysm which required surgical intervention. In the review of similar cases 30% of the total group of patients became symptomatic requiring further intervention. Morgan and Morrell [6] described an arterial injury complicated by a hematoma causing compressive symptoms, which resolved after external compression was removed. They deduced that the presence of a rapidly growing mass (hematoma, pseudo-aneurysm, etc.) by itself can cause compression of the surrounding structures and the application of external compression will only serve to further complicate the condition.

Surgical repair was the treatment of choice in the past, but the location of the VA increases the postoperative morbidity and mortality risk as it requires extensive exposure for direct visualization of the injury. Resorting to open surgical repair was associated with a mortality rate of 6.93% in a retrospective review of VA injuries involving 101 patients (92 penetrating injuries, three iatrogenic, and one blunt trauma). In cases of injury to surrounding structures, failed endovascular intervention, expanding hematoma, airway obstruction, active/uncontrolled bleeding or hemodynamic instability the patient should be considered for immediate surgery [7-8]. In a retrospective study Hatzitheofilou et al. reported a 20% mortality rate in 20 patients who underwent emergency neck exploration following VA injury, with all deaths occurring in unstable patients who were in shock upon arrival [9]. A review article conducted by Inamasu and Guiot reported that surgical intervention is the treatment of choice in cases of aneurysms and inadvertent insertion of a large bore catheter in an artery [10].

In cases where the ligation of VA could not be avoided it is recommended that an angiogram should be performed preoperatively to assess the diameter of both vertebral arteries. A retrospective study following 15 patients up to seven years after unilateral VA ligation has shown that ligation was followed by an uneventful postoperative period -- not associated with neurological deficit -- provided that the diameter of the ligated artery was equal to or less than that of the contralateral VA. One patient underwent bilateral VA ligation on separate occasions, but prior to the ligation of the remaining right VA the blood flow was preserved via bypass graft connecting it to the right external carotid artery [11]. Curtis et al. documented a case of a misplaced CVC in the VA in a patient who required a sternotomy, and after confirmation of the patency of the contralateral VA the injured artery was ligated and the catheter was removed [12]. The patient developed right-sided Horner’s syndrome post-op that improved weeks later.

In comparison, although our patient was critically ill and had required CPR prior to cannulation she remained stable after the inadvertent arterial cannulation. CTA findings showed no extravasation at the site of entry, and she did not show any clinical compressive/obstructive signs. Her general medical and physical condition, advanced age, and hypoplastic contralateral VA made her an unsuitable candidate for surgery. The vascular surgeon and interventional radiologists resorted to endovascular grafting, as a safer and less invasive option, in order to salvage her dominant VA and preserve the posterior circulation.

The introduction of endovascular procedures into medical practice has drastically changed the diagnostic and therapeutic approach to managing vascular injuries, both in the acute and chronic settings. This minimally invasive approach has shown to have an overall favorable outcome with a success rate of 85%-89% and a reduction in the rate of perioperative complications and length of hospital stay. Although it has been associated with neurological complications (4.7%); stroke and transient ischemic attack (2.1% and 2.6% respectively) [13-14].

Several options for endovascular treatments were suggested such as tamponade by temporary balloon occlusion, stent grafting, vascular closure device, and occlusion by embolization [2, 15-19]. Akkan et al. described the option of manual compression under angiographic monitoring in a misplaced CVC in the VA that failed to achieve hemostasis, and resorted to stent grafting. In a separate case, the successful use of stent graft to seal the point of extravasation following inadvertent catheter insertion in the VA was reported, with an intact cerebral circulation on CTA; however, the patient died of septic shock due to an underlying infection during the post-op period [2]. Although embolization is considered as a second endovascular treatment option, successful coil embolization of the injured VA was reported in several cases with uneventful postprocedural neurological deficit, provided that the contralateral flow to the posterior circulation is sufficient [16-18]. In addition, it has been used as an adjunct to open surgery to minimize intraoperative and postoperative bleeding in cases of penetrating VA injury [4, 14].

In our case, permanent occlusion using coil embolization was not considered as the injured vessel was dominant. Instead our interventionist resorted to stent grafting to cover the site of catheter entry. The patient tolerated the procedure well and no complications were noted. Immediately after the procedure she was loaded with plavix 300 mg and aspirin 300 mg; followed by daily plavix 75 mg, aspirin 75 mg, and clexane 40 mg. Postintervention assessment showed anterograde flow of the right VA and a very mild, noncompressive hematoma in the medial aspect of the neck. Furthermore, follow up CT brain showed no evidence of ischemic changes.

## Conclusions

Iatrogenic injury to the VA with CVC is rare, but a high clinical suspicion and immediate management can decrease the rate of morbidity and mortality. In the event of inadvertent arterial cannulation, the catheter should be kept in situ only to be removed once the site of injury is visualized under open surgical exploration or angiographic monitoring, where the option of immediate intervention is available. Although various case reports have mentioned several treatment options, there is no definitive guideline to treat such cases and thus the treatment of choice should aim to be the one that offers minimally invasive options and complete reconstruction of the injured vessel. Endovascular management in such cases is minimally invasive and effective.
